# Renal Involvement in Multisystem Inflammatory Syndrome in Children: Not Only Acute Kidney Injury

**DOI:** 10.3390/children10101661

**Published:** 2023-10-07

**Authors:** Alessandra Meneghel, Valentina Masenello, Fiorenza Alfier, Stefania Giampetruzzi, Camilla Sembenini, Giorgia Martini, Francesca Tirelli, Davide Meneghesso, Francesco Zulian

**Affiliations:** 1 Pediatric Rheumatology Unit, Department for Woman and Child’s Health, University of Padua, 35128 Padua, Italy; 2 Pediatric Nephrology Unit, Department for Woman and Child’s Health, University of Padua, 35128 Padua, Italy

**Keywords:** MISC, SARS-CoV-2, kidney, AKI, tubular dysfunction

## Abstract

Kidney involvement has been poorly investigated in SARS-CoV-2 Multisystem Inflammatory Syndrome in Children (MIS-C). To analyze the spectrum of renal involvement in MIS-C, we performed a single-center retrospective observational study including all MIS-C patients diagnosed at our Pediatric Department between April 2020 and May 2022. Demographic, clinical, pediatric intensive care unit (PICU) admission’s need and laboratory data were collected at onset and after 6 months. Among 55 MIS-C patients enrolled in the study, kidney involvement was present in 20 (36.4%): 13 with acute kidney injury (AKI) and 7 with isolated tubular dysfunction (TD). In eight patients, concomitant AKI and TD was present (AKI-TD). AKI patients needed higher levels of intensive care (PICU: 61.5%, *p* < 0.001; inotropes: 46.2%, *p* = 0.002; second-line immuno-therapy: 53.8%, *p* < 0.001) and showed lower levels of HCO3- (*p* = 0.012), higher inflammatory markers [neutrophils (*p* = 0.092), PCT (*p* = 0.04), IL-6 (*p* = 0.007)] as compared to no-AKI. TD markers showed that isolated TD presented higher levels of HCO3- and lower inflammatory markers than AKI-TD. Our results indicate a combination of both pre-renal and inflammatory damage in the pathogenesis of kidney injury in MIS-C syndrome. We highlight, for the first time, the presence of tubular involvement in MIS-C, providing new insights in the evaluation of kidney involvement and its management in this condition.

## 1. Introduction

Multisystem Inflammatory Syndrome in Children (MIS-C) is a severe hyperinflammatory disease occurring 2–6 weeks after SARS-CoV-2 infection or exposure. This condition was described for the first time in April 2020 by clinicians in the United Kingdom [1]. The Centers for Disease Control and Prevention (CDC) and the World Health Organization (WHO) have subsequently proposed case definition criteria for MIS-C which lay out a clinical picture of persistent fever, elevation of inflammatory markers, evidence of two affected organ systems (e.g., cardiac, gastrointestinal, renal, hematologic, dermatologic and neurologic) with exclusion of other causes. Affected patients are less than 21 years old and present a positive history of a previous COVID-19 infection or exposure [2,3]. Clinical manifestations range from milder symptoms including fever, rashes and gastrointestinal symptoms to life-threatening conditions [4,5]. Although multiple systems can be affected in MIS-C, the cardiovascular one is mostly involved with high rates of cardiogenic shock and PICU admission [4,5].

In adults with primary SARS-CoV-2 infection, renal complications have been widely reported and include acute kidney injury (AKI), proteinuria and hematuria [6]. Up to one quarter of hospitalized infected patients developed AKI, which has been identified as a negative prognostic marker [7]. During acute infection, a direct cytopathic role of SARS-CoV-2 resulting in kidney damage has been hypotized [6], although recent studies have also suggested other pathogenetic mechanisms such as renin–angiotensin–aldosterone system (RAS) imbalance, cytokine storm, endothelial dysfunction and/or hemodynamic instability [8]. Angiotensin-converting enzyme type 2 (ACE2-R), the proposed receptor for SARS-CoV-2, is expressed in the kidney epithelium, especially in proximal tubular cells [9,10]. Few studies have analyzed the characteristics of tubular involvement in SARS-CoV-2 infected adults and children [11,12,13,14]. Kidney biopsy revealed variable degrees of tubular necrosis and, less frequently, glomerular involvement [9]. SARS-CoV-2 detection in urine samples of infected patients have been also described [15], and a study performed on autopsies of 26 COVID-19 patients demonstrated virus particles in the epithelium of proximal tubules and podocytes [10]. These considerations have suggested that kidney damage may be directly induced by the virus during acute infection, although the exact viral cytopathic mechanism actually remains not completely understood.

Kidney involvement in pediatric patients affected by COVID-19 has been poorly described. Few studies reported AKI, nephrotic proteinuria, thrombotic microangiopathic damage, acute tubular necrosis, necrotizing glomerulopathy, minimal change disease and C3 glomerulopathy [9,16,17,18,19]. Very few studies have investigated tubular damage in children with COVID-19 infection [14,16].

Kidney involvement in MIS-C has been even less explored so far. According to a recent systematic review and meta-analysis, up to one fifth of children with MIS-C develops AKI with the need of kidney replacement therapy in 5–37% of cases [20]. Moreover, AKI seems to be a negative prognostic factor since it is associated with higher probability of death and pediatric intensive care unit (PICU) admission [20,21].

The pathogenesis of kidney involvement in MIS-C is still unclear and probably multifactorial. The main proposed mechanisms include a hyperimmune response, renal hypoperfusion due to dehydration and cardiac dysfunction, RAS imbalance, endothelial dysfunction and drug toxicity [22].

In addition, few studies reported the presence of hematuria, proteinuria and pyuria in patients with MIS-C [23,24,25]. To the best of our knowledge, no study has focused so far on tubular dysfunction children with COVID-19-related multisystem inflammatory syndrome.

This study aims to describe the spectrum of kidney involvement in a cohort of MIS-C patients and to discuss possible pathogenetic mechanisms.

## 2. Materials and Methods

We performed a single-center retrospective observational study enrolling all patients diagnosed with MIS-C at the Pediatric Department of Padua between April 2020 and May 2022. Patients were diagnosed according to WHO’s MIS-C case definition [3]. We collected data regarding demographic, clinical, laboratory characteristics and types of treatment at clinical onset and after 6 months.

According to the KDIGO, AKI was defined as the elevation of serum creatinine at least 1.5 times compared to the normal baseline for age and/or reduced urine output at least <0.5 mL/kg/h. Tubular dysfunction (TD) was defined by the elevation of acute TD marker (urinary NAG, uNAG, and urinary creatinine ratio). Polyuria was defined as urinary output > 4 mL/kg/h. Hypophosphatemia was defined according to phosphate range for age indicated by our laboratory standard. We considered a tubular reabsorption phosphate (TRP) value below 80% as an indicator of increased phosphaturia. uNAG values and TRP were analyzed during patient hospitalization based on clinical needs, with different times of evaluation, as shown in Table 1. Proteinuria was evaluated in 24 h urine sample, considering pathological value > 10 mg/Kg/day. Hematuria was considered positive or negative based on urine dipsticks performed on admission.

Gastrointestinal involvement was defined as abdominal pain and/or diarrhea and/or vomiting.

Cardiovascular involvement was defined as systolic dysfunction: hypotension (systolic pressure < 5° percentile for age) and/or left ventricular ejection fraction (LV-EF) reduction < 55% and/or left ventricular global longitudinal strain (LV-GLS) depression < −18 and/or elevation of cardiac markers according to our laboratory reference value (Troponin I > 32 ng/L, Brain Natriuretic Peptide > 100 ng/L).

Therapeutic approaches included intravenous immunoglobulins (IvIg), corticosteroids and acetylsalicylic acid as first-line therapy. Second-line therapy with biological medication (IL-1 receptor antagonist) was chosen in case of severe and/or persistent symptoms. 

Patients were divided into four groups according to the type of kidney involvement: AKI, no-AKI, isolated TD and AKI associated with TD (AKI-TD).

Categorical variables were compared with Fisher’s or Pearson’s χ^2^ test, and continuous variables were compared with Student’s *t*-test.

## 3. Results

In a two-year time, we diagnosed 55 patients with MIS-C at our Pediatric Department: 21 females (38.2%) and 34 males (61.8%). The mean age at diagnosis was 8 years (range 1.2–17.5). Associated comorbidities were described in four patients: Moebius Syndrome with cerebral palsy (1 case), type 1 diabetes (1 case), Gitelman Syndrome (1 case) and PFAPA syndrome (1 case). Table 2 summarized the main clinical characteristics and laboratory profile of our MIS-C cohort. As reported in Table 2, gastrointestinal involvement was present in 78% of the patients with increased fluid losses due to diarrhea and/or vomit in 63%. Cardiovascular involvement was found in 89%, mainly as systolic dysfunction (Troponin elevation in 46% and pathological pro-BNP values in 83%). Mucocutaneous manifestations were detected in 80% of cases, while only five patients (9%) had central nervous system involvement (three encephalopathy with seizures, two headaches).

Kidney involvement was reported in 20 cases: 13 AKI and 7 TD. Interestingly, eight of these patients had both AKI and TD combined. Table 2 describes the tools used to investigate kidney involvement.

Considering the whole MIS-C cohort, AKI incidence was 23% (13/55). All patients presented with AKI on hospital admission, 10/13 showed rapid recovery within three days and none required renal replacement therapy. In three patients, complete resolution required longer time (maximum 6 days). In the AKI group: 11/13 (84.6%) had stage 1 AKI, and 2/13 (15.3%) presented stage 3 AKI. Nephrotic proteinuria (>50 mg/kg/die) was reported in two patients, both presenting with AKI stage 3 and positive u-NAG. u-NAG was tested in only 19 patients, and 15 (78%) had pathological values, of whom 7 (46%) had interestingly isolated TD without AKI. Considering urinary output, polyuria was detected in 16% of patients during hospitalization, five with elevated u-NAG. At 6 months, only 1 case presented sequelae with low-grade hypertension. This patient, affected by Moebius syndrome and cerebral palsy, presented a severe kidney involvement (AKI grade 3) at disease onset, nephrotic proteinuria and needed PICU admission for inotropic support. All the other patients with kidney involvement presented with complete recovery at 6 months. 

No patients presented with macro-hematuria. At the urine dipstick performed on hospital admission we detected micro-hematuria in 8/35 (22%). No patients presented with glycosuria.

No renal biopsies were performed.

In our MIS-C cohort, patients with renal involvement were subdivided into four groups: AKI, No-AKI, TD-AKI, isolated TD.

If we consider patients with AKI and No-AKI, the main difference consisted of a more severe disease course of AKI patients since they more frequently required admission to PICU (61.5%, *p* < 0.001), hemodynamic support with inotropes (46.2%, *p* = 0.002) and second-line treatment with IL-1 inhibitors (53.8%, *p* < 0.001). This clinical evidence was associated with higher inflammatory markers such as neutrophils count (*p* = 0.092), procalcitonin level (*p* = 0.04) and serum IL-6 (*p* = 0.007) (Table 3).

Interestingly, we identified seven patients with isolated-TD, without associated AKI. This group, compared to patients in whom TD was associated with AKI (TD-AKI), presented higher levels of HCO3-, lower inflammatory markers and no need for intensive care or second line therapy with anti-IL 1 inhibitors (Table 4).

Finally, in the whole MIS-C cohort we found a high rate of patients with hypophosphatemia (38/51, 75%; mean value 1.15 mmol/L). Inappropriate phosphaturia, which is a sign of tubular dysfunction, was reported in 20% of tested cases. Hypophosphatemia was associated with higher values of IL6 (*p* = 0.044) and need for inotropic support (*p* = 0.05) (Table 5).

## 4. Discussion

In the pediatric population, AKI has been described with higher frequency in MIS-C patients than in acute SARS-CoV-2 infection [23], with a reported incidence varying between studies. A recent systematic review and meta-analysis stated that up to 20% of patients with MIS-C developed AKI [20]. Consistent with this data, in our study, we found AKI in 23% of patients. In our study, AKI presented a self-limiting course with no patients needing renal replacement therapy and only one presenting with hypertension at 6-month follow-up. Our results are different from other reports, in which renal replacement therapy has been reported in up to 15% of patients with MIS-C and kidney involvement [20,21,22,26,27]. Since in our cohort AKI subgroup presented with a more severe disease course, requiring more frequently intensive care support and second line immunomodulatory therapy, we may suppose that a prompt diagnosis and support with a more aggressive therapeutical approach may have favored a complete recovery in our patients. 

Possible causes of kidney injury in MIS-C include cardiac dysfunction, hypovolemia, cytokine storm, endothelial dysfunction, rhabdomyolysis, and nephrotoxic drugs [8,27,28]. It has been reported that AKI in MIS-C is associated with higher inflammatory markers, greater rates of systolic dysfunction, need of inotropes and lower levels of albumin and bicarbonates, suggesting a prerenal component in the pathogenesis of kidney insufficiency in this syndrome [26,28].

In particular, some Authors have described higher values of inflammatory biomarkers such as white blood cells, CRP, procalcitonine, D-dimer, ferritin and IL-6 in MIS-C patients presenting with AKI. In the same studies association with systolic dysfunction, need of inotropes and lower levels of albumin and bicarbonates have been observed [25,26,28] Therefore, these data suggested a double component in the pathogenesis of kidney injury in MIS-C, due to both an inflammatory pathway and prerenal injury [26]. 

In our study, patients with AKI demonstrated higher degrees of inflammatory markers (neutrophils, PCT, IL-6) and presented with clinical and biochemical data consistent with hypovolemia (history of vomit/diarrhea, lower serum bicarbonates). Our study also confirms that AKI is associated with a more severe course with higher need of intensive care, inotropes, and second-line immunomodulatory therapy with anti-IL1 inhibitors [19,25,28]. Thus, our results support the hypothesis that kidney injury in MIS-C is sustained both by inflammation and hypovolemia, reinforcing the importance of a prompt and appropriate management with hemodynamic support and immunomodulators to prevent the kidney dysfunction. Furthermore, these two mechanisms may be closely related to each other since hyperinflammation may contribute to a capillary leak syndrome worsening the hypovolemic state and the prerenal injury. 

While the available literature on kidney involvement in MIS-C is mostly focused on AKI, a few reports have described acute tubular involvement in pediatric patients during SARS-CoV-2 infection but never during MIS-C [9,14,16]. Devrim at al. described TD in 20.9% of hospitalized children with SARS-CoV-2 infection, excluding those needing intensive care [14]. Tas et al. reported high levels of urinary beta 2-microglobulin and urinary IL-6 during the acute phase of infection in COVID-19-positive children [16].

Furthermore, few authors have reported tubular dysfunction in acute SARS-CoV-2 infection also in adults. Werion et al. detected elevation of B2-microglobulin in 69% of patients affected by COVID-19 and reported defective reabsorption of phosphate in (19%) and uric acid (46%) [13]. Gustavo et al. observed high excretion of calcium, sodium, phosphate and alkaline pH in urine samples of adults infected with SARS-CoV2 suspecting a tubular dysfunction. In this study, tubular markers such as u-NAG were not studied [12]. Fukao et al. analyzed tubular injury markers (U-NAG, L-FABP, u-B2microglobulin, u-alfa-1-microglobuline) in patients with SARS-CoV2 infection without AKI: patients with severe infection (defined as need of oxygen therapy) had significantly higher tubular damage markers and levels of IL-6, suggesting the possible contributing role of cytokine storm in tubular injury [12]. 

We investigated TD because we clinically observed that some children with MIS-C presented an increased urinary output and hypophosphatemia. When tubular markers were investigated, we detected a high frequency of pathological findings (u-NAG). In patients with previous AKI, TD was suspected and investigated a few days after AKI development (Table 1). In patients with isolated TD, we noticed lower rates of hypovolemia and inflammatory markers (IL-6, PCT) and no need for intensive care and second-line immunomodulatory therapy as compared to AKI-TD subset (Table 4). However, considering that tubular cells are very vulnerable to ischemic damage, we might suppose that tubules represent the first kidney component to be early affected in MIS-C patients, although not further evolving into AKI. 

We also noticed a high percentage of patients with serum hypophosphatemia in our MIS-C cohort. TRP was calculated for 23 patients. In 19 patients with hypophosphatemia and contextual evaluation of phosphate excretion, 20% had inappropriately high excretion of phosphate (TRP < 80%). Hypophosphatemia spontaneously resolved after immunomodulatory therapy for MIS-C without the need for phosphate supplementation. 

Low serum levels of phosphate have been already described in critically ill children and has been correlated with PICU hospitalization, ventilation, or malnutrition [29,30,31]. The physiopathology of the phenomenon is still uncertain. In our study, we found that low levels of serum phosphate seem to be not related to tubular dysfunction but more probably to the inflammatory state and a more severe course, given the positive correlation of hypophosphatemia with higher serum levels of IL-6 and use of inotropes (Table 5). 

Considering the cytokines’ profile, in our cohort, we found that MIS-C patients with AKI had significantly higher levels of serum IL-6 (Table 3). In SARS-CoV-2-related conditions, there is growing interest in cytokines profiles and IL-6 has been considered one of the major contributing factors of “cytokine storm” [32], so that in critically ill patients, IL-6 inhibitors have been used as possible targeted-therapy [33]. IL-6 antagonist has been also proposed as rescue therapy in MIS-C patients who did not respond to IL1 inhibitors [34]. The diagnostic and prognostic significance of IL-6 levels in MIS-C is not clearly understood. Experimental studies showed IL-6 activation and secretion by podocytes, endothelial cells, mesangial cells, and tubular epithelial cells in inflammatory diseases with kidney involvement [35]. In animal models with ischemic AKI, increased IL-6 transcription and signaling has been demonstrated both locally and systemically, suggesting IL-6 as a potential biomarker and therapeutic target in ischemic AKI [36]. 

Treatment with IvIg is an already described cause of renal injury, mostly reported in patients with pre-existing renal disease, hypertension, diabetes mellitus, volume depletion and concomitant use of nephrotoxic drugs [37,38,39,40]. Osmotic injury, glomerular precipitation of immune complexes, acute tubular obstruction and renal hypoperfusion have been proposed as possible pathogenetic mechanisms of kidney damage [37]. Renal oliguric dysfunction is the most common manifestation, occurring within 10 days after IvIg infusion, with a maximum increase in serum creatinine on day 5 and complete recovery within 15 days [37,38]. In our AKI cohort, renal dysfunction was already present on admission, before IvIg administration, and none presented with worsening of renal function after the infusion. Based on our experience, patients with isolated-TD presented a positive clinical course and less risk factors for IvIg-related kidney damage, as described above. On the other side, we cannot completely exclude a possible contributing role of IvIg in tubular involvement in this subset of patients. Further studies with larger cohorts are needed to better understand the possible association between TD and exposure to IvIg in MIS-C patients.

Our study presents some limitations. Firstly, it is a single-center retrospective study. Although being monocentric favored uniformity in data collection, the lack of a routinary protocol for tubular markers’ screening in all patients at admission, during active disease and follow-up resulted in small samples not always statistically comparable. Moreover, TD markers’ research was based on clinical needs with a possible selection bias causing overestimation of the rates of pathological results. 

To the best of our knowledge, however, this is the first study to show tubular dysfunction in MIS-C patients, providing new insights in the evaluation of kidney injury in this condition. We suggest evaluating tubular markers in all MIS-C patients at admission and during the active phase of the disease. Our results also confirmed the high prevalence of kidney involvement in MIS-C, especially in patients with a severe course, supporting both pre-renal and inflammatory pathogenetic causes for AKI in this syndrome.

## Figures and Tables

**Table 1:** Tools to evaluate kidney involvement.

	Data Available (n)	Pathological Values (n)	Time of Detection
**AKI evaluation**			
Creatinine and/or oliguria	55	13/55 (23%)	On admission
**TD evaluation**			
u-NAG	19/55	15/19 (55%)	2–17 * days (median: 6)
Hypophospatemia	51/55	38/51 (75%)	1–10 * days (median: 2 days)
TRP	23/55	4/23 (17%)	1–10 * days (median: 2 days)
Polyuria	55/55	723/55(14%)	During hospitalization course
**Other kidney involvement**			
24 h Proteinuria	26	9/26 (34%) (2 nephrotic, 7 non nephrotic)	During hospitalization course
Hematuria	35	8/35 (22%)	On hospital admission

**Legend:** AKI: acute kidney injury; TD: tabular damage; TRP: tubular reabsorption phosphate; u-NAG: urinary N-acetyl-beta-D-glucosaminidase; * days between MIS-C diagnosis and laboratory test.

**Table 2:** Clinical characteristics and laboratory findings of MIS-C cohort.

Variables	N (tot)	N (Frequence/Median), [IQR]
Male, n (%)	55	34 (61.8)
Age, yr (range)	55	8 (1.2 to 17.5)
Comorbidities (%)	55	5 (9)
**Presenting symptoms, n (%)**		
Gastrointestinal	55	48 (87.3)
Fluid losses	55	35 (63.5)
Abdominal pain	55	43 (78.1)
Cardiovascular	55	49 (89.1)
Systolic dysfunction	44	36 (81.8)
Mucocutaneous	55	44 (80)
Central Nervous System	55	5 (9.1)
Kidney involvement	55	20 (36.6)
AKI	55	13 (23)
Tubulopathy	19	15 (78.9)
AKI-TD	15	8 (53.3)
TD	15	7 (46.6)
Nephrotic proteinuria	13	2 (15.8)
Non nephrotic proteinuria	13	7 (53.8)
Microhematuria	35	8 (22)
Polyuria	42	7 (16)
**Admission parameters**		
WBC (cells/mmc)	55	8810 [6825–12,895]
Neutrophils (cells/mmc)	55	7100 [4100–9165]
PLT (cells/mmc)	55	159,000 [104,500–244,500]
CRP (mg/L)	55	138 [90.25–210]
PCT (ng/mL)	45	4.8 [0.69–27]
IL-1A (ng/L)	36	2 [2–2.4]
IL-1B (ng/L)	36	6 [5–12.5]
IL-6 (ng/L)	36	46 [15.5–262]
Urea (mmol/L)	53	4.3 [3.15–6.91]
Creatinina (umol/L)	53	43 [34–63]
Albumin (mg/lL)	55	29.5 [27–34.75]
Phosphate (mmol/L)	51	1.15 [0.88–1.41]
Bicarbonates (meq/L)	23	22 [20.6–23.85]
Troponin (ng/L)	49	34 [8.8–99.5]
NT-pro-BNP (ng/L)	47	225.5 [126.75–922.75]
TRP < 80 (%)	23	4 (17.3)

**Legend.** AKI: acute kidney injury; TD: tubular dysfunction; WBC: white blood cells; PLT: platelet; CRP: C-reactive protein; PCT: procalcitonin, IL: interleukin; NT-pro-BNP: amino terminal fragment of brain-type natriuretic peptide; TRP: phosphate tubular reabsorption.

**Table 3:** Clinical characteristics and laboratory profile in AKI and No-AKI patients.

Variables	AKI Frequency Mean Value	No-AKI Frequency Mean Value	*p*
*Clinical profile*			
Male, n	9/13 (69.2%)	25/42 (59.5%)	0.43
Comorbidities	1/13 (7.7%)	4/42 (10.5%)	0.99
Nephrotoxic medications	4/13 (30.8%)	1/42 (2.4%)	**0.013**
Fluid losses	11/13 (84.6%)	24/42 (57%)	0.095
Systolic dysfunction	10/11 (90.9%)	26/33 (78.8%)	0.64
Tubular Damage	8/9 (88.9%)	7/10 (70%)	0.58
Inotropes	6/13 (46.2%)	3/42 (7.1%)	**0.002**
PICU admission	8/13 (61.5%)	2/38 (5.3%)	**<0.001**
IL-1 inhibitor	7/13 (53.8%)	2/42 (4.8%)	**<0.001**
Polyuria	4/13 (30.7%)	3/36	0.070
*Laboratory profile*			
Neutrophils (cells/mmc)	11,054.62	9175.95	0.092
Platelets (cells/mmc)	156,230	202,618	0.22
CRP (mg/L)	189.68	147	0.33
Procalcitonine (ng/mL)	127.82	51.44	**0.04**
IL-6 (ng/L)	294.76	63.18	**0.007**
IL-1a (ng/L)	15	2	0.076
IL-1b (ng/L)	20	13	0.55
D-dimer (ug/mL)	2828.23	1478.16	**<0.001**
Albumin (mg/L)	30.15	30.3	0.92
CPK (U/L)	256.85	181.64	0.60

**Legend.** PICU: pediatric intensive care unit; IL: interleukin; CRP: C-reactive protein; IL: Interleukin; CPK: creatine phosphokinase.

**Table 4:** Clinical characteristics and laboratory profile of MIS-C patients with tubular disease.

	AKI-TD (n = 8) Frequency/Mean Value	Isolated TD (n = 7) Frequency/Mean Value
Fluid losses	7/8 (87.5%)	6/7 (85%)
PICU admission	7/8 (87.5%)	0/7 (0%)
IL-1 inhibitor	6/8 (75%)	0/7 (0%)
Inotropes need	5/8 (62%)	0/7 (0%)
Nephrotic proteinuria	2/6 (33%)	0/4 (0%)
PCT (ng/mL)	180	22
CRP (mg/L)	220	153
Neutrophils (cells/mmc)	11,251	6594
IL-6 (ng/L)	367	131
IL-1a (ng/L)	2	2
IL-1b (ng/L)	10	9
Bicarbonates (mq/L)	15	23

**Legend.** PICU: pediatric intensive care unit; PCT: procalcitonin; CRP: C-reactive protein; IL: Interleukin.

**Table 5:** Main clinical and laboratory findings in patients with and without hypophosphatemia.

	Hypophosphatemia (n = 38) Frequency	No Hypophosphatemia (n = 13) Frequency	*p*
AKI	10/38 (26.3%)	3/13 (23.1%)	1
TD	10/14 (71.4%)	5/5 (100%)	0.5
TRP	4/20 (20%)	0/3 (0%)	1
Inotropes need	9/38 (23.7%)	0/15 (0%)	**0.05**
IL-1 inhibitor	8/38 (21%)	1/15 (7.6%)	0.41
PCT (ng/mL)	84	12	0.051
CRP (mg/L)	155.5	155.3	0.88
IL-6 (ng/L)	148	20	**0.044**
D-dimer (ug/mL)	2357	891	0.12

**Legend.** AKI: acute kidney injury; TD: tubular dysfunction; TRP: phosphate tubular reabsorption; IL: interleukin; PCT: procalcitonin; CRP: C-reactive protein.

## Data Availability

The data presented in this study are available on request from the corresponding author. The data are not publicly available due to privacy restrictions.
